# CDT of *Clostridioides difficile* Induces MLC-Dependent Intestinal Barrier Dysfunction in HT-29/B6 Epithelial Cell Monolayers

**DOI:** 10.3390/toxins15010054

**Published:** 2023-01-07

**Authors:** Lucas Heils, Martina Schneemann, Ralf Gerhard, Jörg-Dieter Schulzke, Roland Bücker

**Affiliations:** 1 Clinical Physiology, Charité—Universitätsmedizin Berlin, Campus Benjamin Franklin, 12203 Berlin, Germany; 2 Institute of Toxicology, Hannover Medical School, 30625 Hannover, Germany

**Keywords:** binary toxin, tight junction, barrier function, claudin, tricellulin, occludin, cytoskeleton, actin, leak flux, STED microscopy

## Abstract

Background: *Clostridioides difficile* binary toxin (CDT) defines the hypervirulence of strains in nosocomial antibiotic-induced colitis with the highest mortality. The objective of our study was to investigate the impact of CDT on the intestinal epithelial barrier and to enlighten the underlying molecular mechanisms. Methods: Functional measurements of epithelial barrier function by macromolecular permeability and electrophysiology were performed in human intestinal HT-29/B6 cell monolayers. Molecular analysis of the spatial distribution of tight junction protein and cytoskeleton was performed by super-resolution STED microscopy. Results: Sublethal concentrations of CDT-induced barrier dysfunction with decreased TER and increased permeability for 332 Da fluorescein and 4 kDa FITC-dextran. The molecular correlate to the functional barrier defect by CDT was found to be a tight junction protein subcellular redistribution with tricellulin, occludin, and claudin-4 off the tight junction domain. This redistribution was shown to be MLCK-dependent. Conclusions: CDT compromised epithelial barrier function in a human intestinal colonic cell model, even in sublethal concentrations, pointing to barrier dysfunction in the intestine and leak flux induction as a diarrheal mechanism. However, this cannot be attributed to the appearance of apoptosis and necrosis, but rather to an opening of the paracellular leak pathway as the result of epithelial tight junction alterations.

## 1. Introduction

*Clostridioides difficile* (*C. difficile*) is a Gram-positive, sporulating, and anaerobic bacterium and a frequent cause of nosocomial infections [1,2]. *C. difficile* infection is the most common cause of antibiotic-associated colitis. The patients present with severe diarrhea, potential development of pseudomembranous colitis, and the formation of toxic megacolon [3]. The main virulence factors produced by *C. difficile* are the glycosylating toxins TcdA and TcdB, and additionally in hypervirulent *C. difficile* strains, the ADP-ribosylating toxin CDT. The presence of this CDT toxin is associated with increased lethality, indicating additive pathomechanisms. Thus, the presence of the binary toxin increases the risk for severe disease progression, complications, recurrence of infection, and mortality [4,5]. The underlying mechanisms of hypervirulence remain unclear. CDT belongs to the family of binary toxins with two subunits: CDTa and CDTb. CDTb uses LSR (lipolysis-stimulated lipoprotein receptor, syn. angulin-1) for receptor binding to the host cell and mediates the cellular uptake of CDTa [6]. Internalized CDTa causes the ADP-ribosylation of G-actin, thereby inhibiting the polymerization of F-actin [7]. CDT has been shown to induce cell rounding, loss of cell viability, and microtubule redistribution [8]. Moreover, CDT affects the proteome and, in particular, the phosphorylation status of cellular proteins, including increased phosphorylation of calmodulin kinase motifs, which contributes to the regulation of myosin light chain (MLC) and MLC kinase (MLCK) activity [9,10]. The cytoskeletal integrity is decisive for intestinal epithelial barrier function via regulation of tight junction (TJ) proteins and is supposed to be targeted by CDT during *C. difficile* infection. The intestinal TJ in man consists of 26 claudins, occludin, tricellulin, and further accessory TJ proteins maintaining the barrier function in different intestinal segments, depending on their composition of claudins with barrier-forming or pore-forming properties. The action of *C. difficile* toxins on barrier function was originally shown with respect to occludin, but not for barrier-forming claudins [11] or the tricellular TJ (tTJ), the latter of which provides, if disrupted, a pathway for macromolecules through the epithelial barrier [12,13]. Changes in the subcellular distribution of intestinal TJ proteins can cause diarrhea by a leak flux mechanism and a paracellular loss of solutes and water into the lumen. Furthermore, this TJ redistribution can induce the leaky gut phenomenon, the paracellular uptake of luminal antigens with the consequence of an immune-mediated self-enforcing barrier disruption [14,15].

Thus, the aim of the present study was to identify the epithelial barrier pathology with regard to TJ alterations in intestinal epithelial cells in response to CDT and to enlighten the inherent molecular pathomechanisms for a better understanding of its pathology in the human colon.

## 2. Results

### 2.1. CDT Impairs Epithelial Barrier Integrity of HT-29/B6 Monolayers

**Figure 1:**
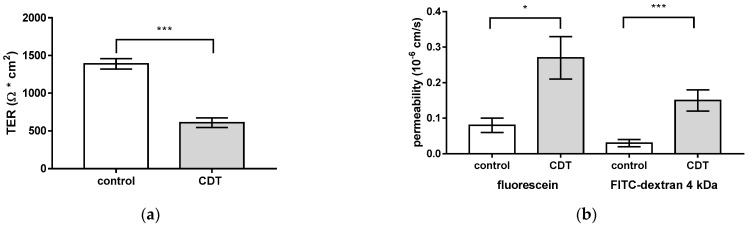
Epithelial barrier function in human HT-29/B6 epithelial cell monolayers 24 h after *Clostridioides difficile* binary toxin (CDT) treatment. (**a**) Functional characterization of the effect of CDT on the intestinal epithelial cells with measurements of transepithelial electrical resistance (TER) and (**b**) macromolecular fluxes of the paracellular markers 332 Da fluorescein and 4 kDa FITC-dextran. CDT was applied from the apical side with a concentration of 5 ng/mL CDTa together with 10 ng/mL CDTb. * *p* < 0.05, *** *p* < 0.001, Student’s *t*-test, n = 5.

In order to investigate the impact of CDT on the epithelial barrier, human colon carcinoma HT-29/B6 cell monolayers [16] were treated with 5 ng/mL CDTa and 10 ng/mL CDTb. After 24 h, the transepithelial electrical resistance (TER) in CDT-treated monolayers was reduced by half compared to control monolayers, indicating impaired integrity of the paracellular pathway (Figure 1a). Concomitantly, treatment with CDT tripled the paracellular permeability for the macromolecule marker fluorescein (332 Da) and increased the permeability for fluorescein isothiocyanate (FITC)-dextran (4 kDa) by a factor of 5.5 (Figure 1b). Treatment with the individual subunits of CDT in a concentration of 5 ng/mL CDTa and 10 ng/mL CDTb resulted in an unchanged TER after 24 h. With a higher concentration of 3000 ng/mL, CDTb reduced the TER (41 ± 9% of initial TER after 24 h, *p* < 0.01, n = 4), whereas CDTa had no effect on TER (102 ± 2% of initial TER value after 24 h, n.s., n = 3).

### 2.2. Necrosis Induction and the Rate of Apoptotic Epithelial Cells Are Unchanged after Treatment with Sublethal Concentrations of CDT

**Figure 2:**
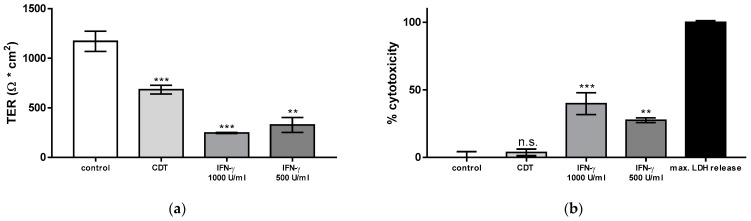
Barrier function and cytotoxicity in HT-29/B6 intestinal cell monolayers. (**a**) Transepithelial electrical resistance (TER) in epithelial cell monolayers after 24 h of treatment with CDT from the apical side with a concentration of 5 ng/mL. In these experiments, 5 ng/mL CDTa was applied together with 10 ng/mL CDTb. In parallel experiments, treatment with 500 and 1000 U/mL interferon gamma (IFN-γ) as a positive control for induction of necrosis decreased TER after 72 h. (**b**) A lactate dehydrogenase (LDH) release assay was used to assess necrotic cell death. The negative control monolayers remained untreated. The positive control monolayers with maximum LDH release and 100% necrosis induction were treated with a detergent lysis buffer. n.s. = not significant, ** *p* < 0.01, *** *p* < 0.001, one-way ANOVA with Bonferroni correction, n = 3–5.

**Figure 3:**
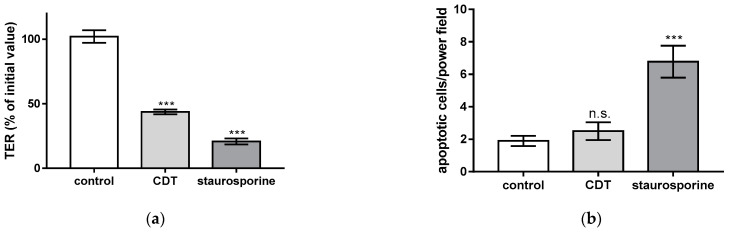
Barrier function and apoptotic cell death rate in HT-29/B6 cell monolayers. (**a**) Transepithelial electrical resistance (TER) in epithelial cell monolayers after 24 h of treatment with CDT from the apical side (5 ng/mL CDTa and 10 ng/mL CDTb) or after 24 h of treatment with 1 µM staurosporine. (**b**) A terminal deoxynucleotidyl transferase dUTP nick-end labeling (TUNEL) assay was used to visualize fragmented target cell DNA as a sign of apoptosis induction. TUNEL-positive cells were counted microscopically. Cell death induction was expressed as TUNEL-positive cells after CDT or staurosporine treatment. n.s. = not significant, *** *p* < 0.001, one-way ANOVA with Bonferroni adjustment, n = 5–10.

Next, the contribution of cytotoxicity to CDT-induced epithelial barrier defect was examined. As a positive control of cytotoxicity, interferon gamma (IFN-γ) as an inductor of necrosis was applied to the cell monolayers, in order to analyze the effect of cell loss in the monolayers on barrier dysfunction (Figure 2). In CDT-treated cell monolayers, no correlation was found between barrier dysfunction and cytotoxicity, since sublethal CDT concentration as used here did not induce necrosis. Thus, barrier-relevant epithelial cell death was not increased by CDT treatment. In contrast, as a positive control, IFN-γ-treated cells showed an increased LDH release, indicating the induction of barrier-relevant necrosis. Furthermore, in parallel experiments with sublethal CDT concentrations, there was also no evidence for the induction of apoptosis. In contrast, staurosporin, as a positive control for apoptosis induction reduced TER, accompanied by epithelial cell death (Figure 3). In line with this observation, disinhibition experiments with the pan-caspase inhibitor Q-VD-OPh (10 µM) failed to ameliorate the barrier dysfunction induced by CDT (46 ± 2% of the initial TER in CDT-treated monolayers versus 44 ± 2% in CDT plus Q-VD-OPh-treated monolayers, n.s., *p* = 0.41, Student’s *t*-test, n = 5). Thus, the barrier defect of sublethal CDT concentrations has to be attributed to paracellular TJ effects and not to cell death phenomena.

### 2.3. Tight Junction Protein Expression after 24 h of Low-Dose CDT Treatment in HT-29/B6 Monolayers

**Figure 4:**
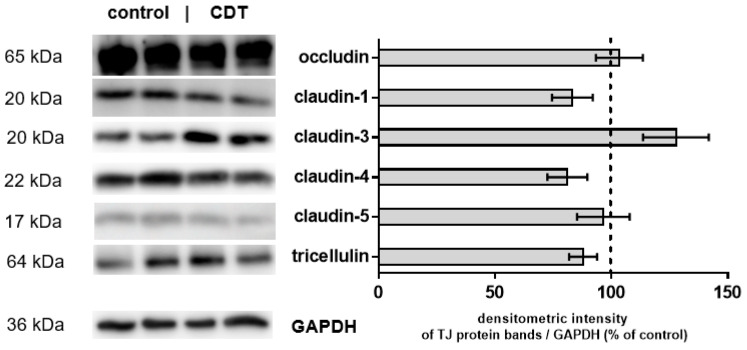
Expression of tight junction proteins in CDT-treated HT-29/B6 monolayers. Western blot was performed on the cell lysates 24 h after exposure to CDT. This representative Western blot shows two lanes of each condition. The expression level of tight junction proteins was normalized with glyceraldehyde 3-phosphate dehydrogenase (GAPDH) expression. Immunoblots were analyzed by densitometry and normalized to the level of GAPDH as a loading control. The dashed line represents the control value set to 100%. The values were not different from controls. n = 4–6 and Student’s *t*-test with Bonferroni adjustment was used for multiple comparisons.

Another reason for the functional impairment of the epithelial barrier could be thedownregulation of barrier-forming TJ proteins. The densitometric analysis of Western blots of the TJ proteins occludin, tricellulin, and claudin-1, -3, -4, -5 from HT-29/B6 monolayers 24 h after treatment with CDT revealed no change in protein expression (Figure 4).

### 2.4. Microstructural Analysis by Super-Resolution Microscopy of CDT Impact on the Tight Junction

**Figure 5:**
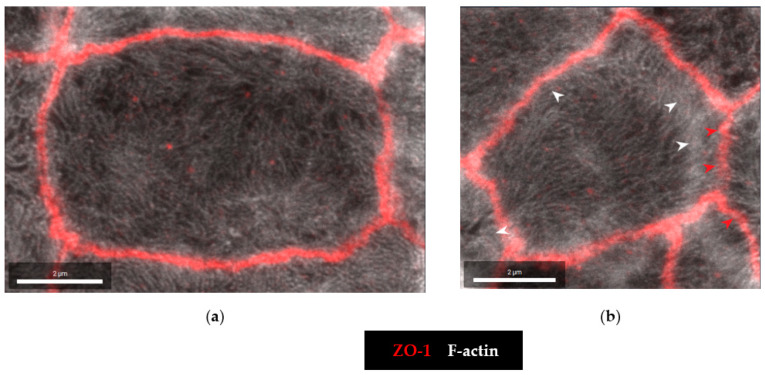
Super-resolution STED microscopy of confluent human HT-29/B6 cells. Microscopic signals of Zonula occludens protein-1 (ZO-1) are shown in red by immunofluorescent staining. F-actin is shown in white by fluorescent phalloidin staining. (**a**) Untreated control cell. (**b**) CDT-treated cell 24 h after exposure to 5 ng/mL CDTa and 10 ng/mL CDTb. White arrowheads indicate perijunctional condensation of F-actin and red arrowheads indicate the onset of TJ ruffling. Bar = 2 µm.

**Figure 6:**
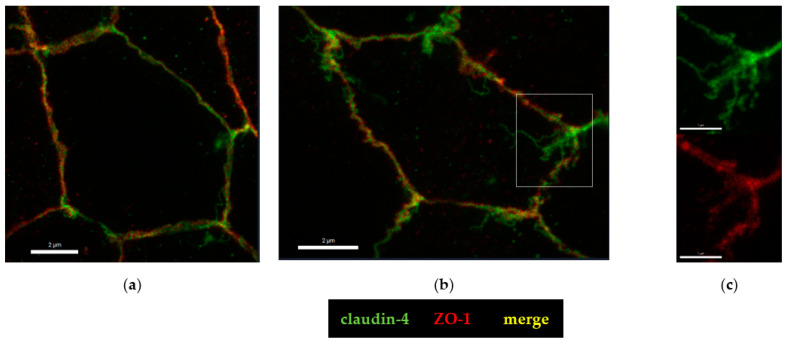
Super-resolution analysis of tight junction proteins with STED microscopy. Immunofluorescence staining colored claudin-4 are green and Zonula occludens protein-1 (ZO-1) are red. (**a**) As a control, untreated HT-29/B6 epithelial cell monolayers were used; bar = 2 µm. (**b**) CDT-treated monolayers after 24 h; bar = 2 µm. (**c**) CDT-treated monolayers and detail of tricellular tight junction, which is enlarged from the white square in image b; bar = 1 µm.

To further analyze the molecular correlate to the functional barrier defect after CDT treatment, we analyzed our HT-29/B6 cell monolayers by super-resolution stimulated emission depletion (STED) microscopy. In untreated controls, the shape of the TJ, here represented by zonula occludens protein-1 (ZO-1), appears smooth, without any discontinuities or ruffling and the cytoskeletal F-actin pattern shows an even distribution of filaments around the perijunctional ring (Figure 5a). In the CDT-treated cells, ruffling of ZO-1 and perijunctional condensation of F-actin around the TJs were observed, without any obvious changes in the F-actin signals throughout the cell, reflecting an intact cytoskeletal organization (Figure 5b). As one abundant representative of the barrier-forming claudin family, claudin-4 was analyzed in more detail in super-resolution images and was found to be frayed and pulled out of the TJ (Figure 6), and was redistributed into mesh-like strand formations at lateral membranes and the tricellular TJ domain. This kind of fraying of TJ proteins was seen in the bicellular TJ but was also prominent in the tricellular TJ domain (Figure 6b,c).

### 2.5. Tricellular Tight Junction Delocalization in CDT-Treated Epithelial Cells

**Figure 7:**
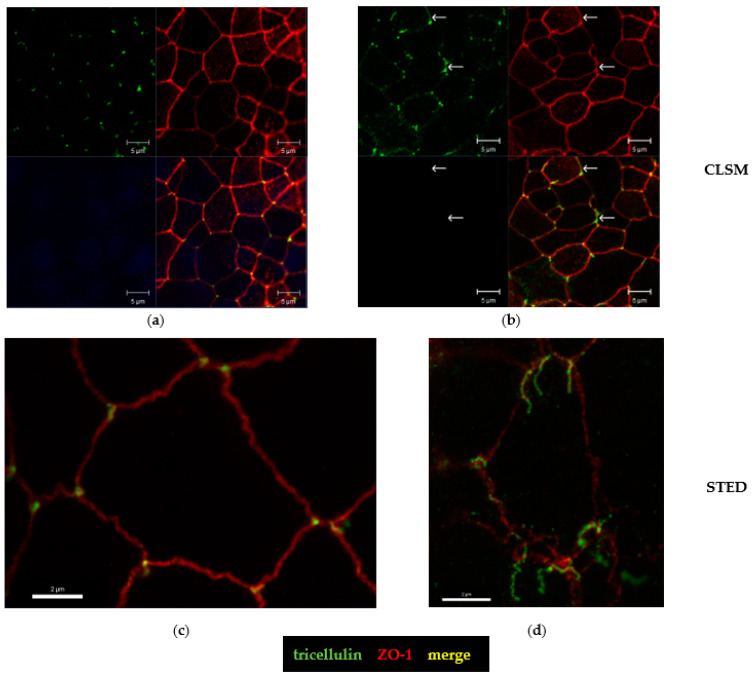
Confocal microscopy and STED microscopy reveal subcellular tricellulin redistribution. The subcellular distribution of the tricellular tight junction protein tricellulin is changed after CDT treatment. Using immunofluorescence staining, colored tricellulin appears green and ZO-1 red. Nuclei are colored blue with 4′-6-diamidino-2-phenylindole (DAPI). (**a**) Conventional confocal laser-scanning microscopy (CLSM) of an untreated control monolayer of human HT-29/B6 epithelial cells; green = tricellulin, red = ZO-1, blue/black = the fluorescence channel for showing the absence of cell nuclei at this cell level, yellow = a merge of green and red. (**b**) CLSM of tricellulin, which is redistributed off the tricellular tight junction domain in CDT-treated cell monolayers. White arrows indicate tricellulin redistribution from tricellular to bicellular TJs; bar = 5 µm. (**c**) STED microscopy of an untreated control cell. (**d**) STED micrograph of a CDT-treated cell; bar = 2 µm.

**Figure 8:**
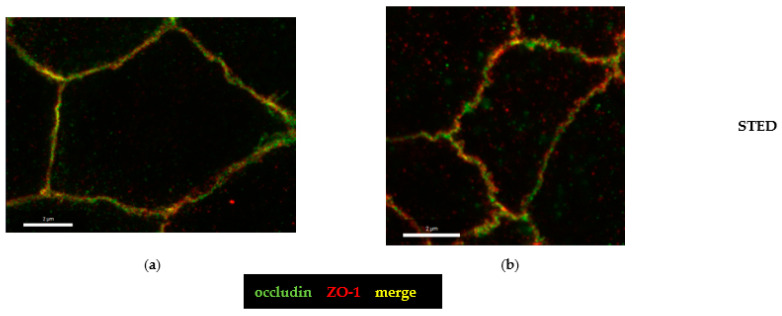
Confocal microscopy and STED microscopy of occludin changes. Occludin is colored green and ZO-1 is colored red by immunofluorescence staining. (**a**) STED microscopy of an untreated control. (**b**) STED of a CDT-treated cell. Occludin signal appears ruffled along the bicellular tight junction domain; bar = 2 µm.

The hint for an involvement of the tricellular tight junction (tTJ) arose from the analysis of claudin-4 staining (Figure 6) and pushed our study toward a more detailed characterization of the tTJ. Immunostainings of CDT-treated HT-29/B6 monolayers revealed already in conventional confocal laser-scanning microscopy (CLSM, Figure 7a,b) a subcellular redistribution of the tTJ protein tricellulin into the bicellular tight junction (bTJ) and to a smaller extent of the TJ domain into intracellular compartments (Figure 7b). Using the higher resolution of STED microscopy, the details of tricellulin redistribution became clearer. In controls, tricellulin was localized with sharp punctated signals at the tTJ (Figure 7c). After CDT treatment, the boundary of tricellulin signaling was no longer sharp and resembled a lining up of tricellulin proteins along the bicellular tight junction with loss of exclusive tricellular localization (Figure 7d), and thus could be responsible for a loss of epithelial barrier function through the tTJ for small macromolecules, such as fluorescein and 4 kDa dextran (Figure 1b). Since occludin, as one member of the tight junction-associated MARVEL protein family (TAMPs), is closely related to tricellulin, which both share the barrier function for the prevention of macromolecular influx [17], we investigated the spatial distribution of occludin in CDT-treated cell monolayers (Figure 8). In STED micrographs, another microstructural pathology of CDT became visible in the bTJ, namely the occludin signal that was ruffled compared to the smooth TJs in the control cell monolayers (Figure 8a,b).

### 2.6. Disinhibition of the Barrier Defect of CDT by Specific Myosin Light-Chain Kinase Inhibitor PIK

**Figure 9:**
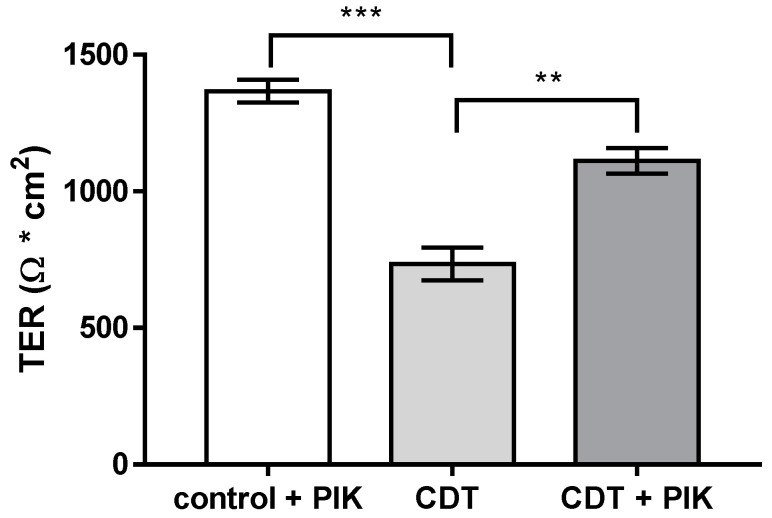
Recovery of the barrier defect induced by CDT (5 ng/mL CDTa and 10 ng/mL CDTb) with 200 µM D-reverse PIK (PIK), a specific MLCK inhibitor [20]. Transepithelial electrical resistance (TER) was measured in HT-29/B6 cell monolayers 24 h after treatment. ** *p* < 0.01, *** *p* < 0.001, n = 5, Student’s *t*-test with Bonferroni correction.

**Figure 10:**
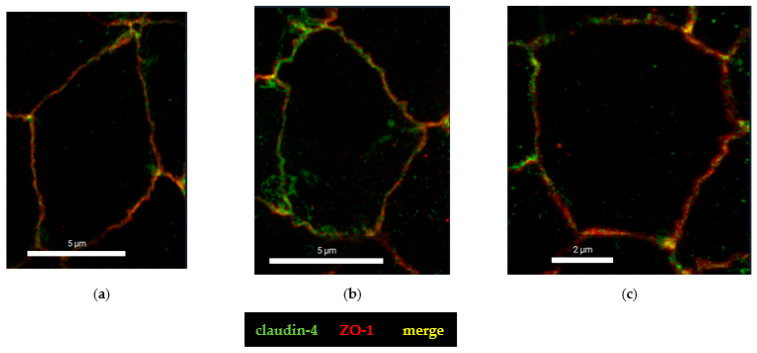
Recovery of paracellular integrity of claudin-4 after CDT treatment and protective treatment with 200 µM D-reverse PIK (PIK). Immunostainings of claudin-4 (green) and ZO-1 (red) in HT-29/B6 cell monolayers after 24 h of treatment, visualized by super-resolution STED microscopy. (**a**) Untreated control; bar = 5 µm. (**b**) CDT treatment; bar = 5 µm. (**c**) Treatment of CDT and PIK; bar = 2 µm.

CDT has been shown to destabilize the actin cytoskeleton, leading to cell death at high concentrations, but in addition, it is able to alter the proteome and the phospho-status of CDT-intoxicated cells [9]. Since we observed ruffling of TJ proteins (Figure 5, Figure 6, and Figure 8) in CDT-treated cells with sublethal concentrations, which indicates a tension of the TJ-connected perijunctional cytoskeleton, we aimed to interfere experimentally with the suspected actin–myosin constriction as a regulator of TJ proteins. Such a regulatory mechanism has already been reported before to be responsible for TJ protein redistribution and barrier function changes in other pathologies [18,19]. Using an inhibitor for the MLCK, the pseudosubstrate PIK, which specifically blocks MLC phosphorylation by the MLCK [20], we could show that the barrier-opening effect of CDT was ameliorated by PIK (Figure 9). Moreover, we could visualize this functional recovery of barrier function at the molecular level by STED microscopy addressing the barrier-forming claudin-4 (Figure 10). PIK restored both the smooth ZO-1 profile and the claudin-4 distribution (Figure 10c).

### 2.7. Lipolysis-Stimulated Lipoprotein Receptor (LSR) Dependence of Barrier Defect of CDT

**Figure 11:**
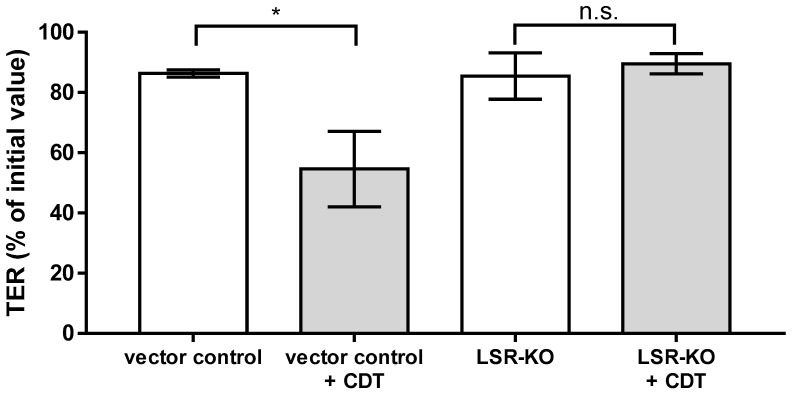
LSR dependence of the barrier defect induced by CDT (5 ng/mL CDTa and 10 ng/mL CDTb) in human HT-29/B6-LSR knockout (LSR-KO) cell monolayers versus vector control monolayers. Transepithelial electrical resistance (TER) was measured 24 h after treatment. * *p* < 0.05, n.s. = not significant. n = 3, Student’s *t*-test.

LSR has been shown to be the receptor facilitating the cellular uptake of CDT [20]. Using HT-29/B6 cell monolayers with an LSR knockout, we were able to show that the presence of LSR is required for the induction of a barrier defect by CDT. In the LSR knockout cells, CDT in a sublethal concentration had no effect on the TER (Figure 11).

## 3. Discussion

The major virulence factors of *C. difficile* are the large glycosylating toxins TcdA and TcdB. However, a significant proportion of *C. difficile* infection strains (5–30%), such as RT027, additionally produce the binary toxin CDT. With its subunits CDTa and CDTb [21], it resembles other bacterial toxins from the binary toxin family, such as *Clostridium perfringens* iota toxin or the *C. botulinum* C2 toxin [22]. In high concentrations, CDT exhibits relevant cytotoxicity, leading to depolymerization of the actin cytoskeleton and rounding of cells [7,23], as well as induction of apoptosis [24]. In contrast, in sublytic concentrations, neither cytotoxic nor cytopathic effects were observed. Instead, we observed a slow weakening of the epithelial barrier over several hours, functionally reflected in a decrease in TER and an increase in the permeability of small macromolecules. We used low (sublytic) concentrations because antibiotic-dependent *C. difficile*-positive diarrhea is not regularly associated with severe pseudomembranous colitis, but often only with low-grade inflammatory responses of the colonic mucosa, which we wanted to study with this experimental design.

Protein expression levels of TJ proteins claudin-1, -3, -4, -5, occludin, and tricellulin, as obtained from Western blots, were unchanged in our HT-29/B6 cell culture model after exposure to sublytic CDT concentrations over 24 h. However, the localization of claudin-4 in the TJ domain of the cells was changed after CDT treatment, visualized by super-resolution microscopy. Claudin-4 was used for this analysis since it is one of the most abundant TJ proteins in the bTJ of the intestine and it has a barrier-maintaining function, i.e., it seals the TJ strands, as indicated by TER and paracellular flux measurements [25]. Furthermore, claudin-4 stands also as a representative for other barrier-forming proteins of the claudin family. Indeed, the altered appearance of claudin-4 gave us the first clue to the molecular correlate of the change in barrier function induced by CDT. Claudin-4 appeared frayed and was pulled out of the TJ and redistributed into mesh-like strand formations at lateral membranes and the tricellular TJ. Fraying may be due to impacts of force emanating from the cytoskeleton, finally leading to subcellular protein redistribution.

However, the most-studied TJ protein regarding barrier function is occludin. Its barrier properties have been rather assigned to the limitation of macromolecule translocation than to ion permeation. This could, e.g., be achieved by stabilizing the localization of other proteins as tricellulin in the tTJ domain of the cells during the highly dynamic process of assembly and disassembly of TJ proteins in the plasma membrane [26]. Thus, the changes in occludin expression in some of the cells of the monolayers observed after CDT treatment could indeed increase macromolecule permeability in the tTJ along this protein distribution dynamics. This contributes to the influx of macromolecules acting as antigens from the intestinal lumen into the mucosa, which is discussed as the leaky gut phenomenon during intestinal inflammation [27,28].

Even more direct evidence for the source of the epithelial barrier defect to macromolecules after CDT treatment comes from our data on tricellulin, which was redistributed off of the tTJ toward the bTJ or into intracellular compartments with a loss of a sharp and exclusive restriction to the tTJ. The consequence is an overall weakening of the epithelial barrier function against luminal macromolecules and antigens [29]. This phenomenon can be interpreted as an additional mechanism in the pathology of hypervirulent *C. difficile* strains, thereby enabling the access of other bacterial toxins, such as the large toxins TcdA and TcdB. Another mechanism of hypervirulence would be an altered toxin expression and regulation in CDT-positive strains [30], even if we have demonstrated in our present study that CDT can induce epithelial barrier dysfunction even at very low and sublethal toxin concentrations. Thus, we hypothesize that the CDT-dependent facilitated access of TcdA and TcdB to the basolaterally localized receptors Frizzled and CSPG4 is the more relevant role of CDT [31,32]. In contrast to toxins TcdA and TcdB, the receptors of which are localized basolaterally on enterocytes, LSR (syn. angulin-1), the receptor for CDT [33], has an apical localization at the tTJ [34]. This mutual opening of the epithelial barrier by toxins with different receptor localization might indeed be a novel explanation for additive toxin effects and the pronounced severity of infection with CDT-positive stains, although this hypothesis requires further experimental confirmation in the future.

Furthermore, as a consequence of these mutual toxin effects, one has to take into account the facilitated access of toxins to the mucosal subepithelial immune cells, as a result of which intestinal inflammatory processes are intensified along the leaky gut concept. It has been argued that such effects on mucosal immune cells in *C. difficile* infection lead to a more severe disease outcome [35,36]. Furthermore, it may be reasonable to conclude that the combined cytotoxicity of the different toxins in CDT-positive strains induces cell death in the epithelial cell layer such as apoptosis, necrosis, necroptosis, or autophagy. Although clinical isolates of TcdA^−^/TcdB^−^/CDT^+^ strains of *C. difficile* were described to be associated with specific, non-severe forms of CDI, the cytotoxic and pathogenic effect of CDT alone is still under discussion [37,38]. In this context, the ability of the pore-forming CDTb subunit to induce calcium influx into epithelial cells should be mentioned [23,39]. Calcium influx caused by other pore-forming toxins, such as AerA from *Aeromonas hydrophila* or HlyA from B2 *Escherichia coli*, can beyond cell death induction, mediate disruption of the intestinal TJ via activation of the MLCK [19,40].

The regulation of the MLCK depends on the intracellular calcium concentration. Higher calcium concentrations activate the MLCK [10]. This induces MLC phosphorylation and leads to constriction of the perijunctional actomyosin cytoskeleton (syn. cortical actin filaments), as a result of which accessory TJ proteins, such as ZO-1, but also strand-forming TJ proteins, such as claudins, occludin, and tricellulin, are sorted out of the TJ domain of the cell. Noteworthy, CDTb alone at sublethal concentrations of 10 ng/mL had no such effect on the TER in our intestinal cell culture model. Only higher concentrations affected TER by pore formation in the apical membrane.

However, there is another CDT-dependent regulatory input. CDT also alters the phosphorylation status of the host cells. It has been recently shown that the target sequences of the calmodulin kinase are more phosphorylated after CDT treatment in HEp-2 cells [9]. This has relevance for the regulation of the MLCK since the MLCK can be activated by phosphorylation, which is mediated by the calmodulin kinase [10]. Thus, subsequent regulatory inputs via MLC and the perijunctional actomyosin cytoskeleton on TJ regulation have to be assumed for CDT. Interestingly, this resembles toxin-dependent TJ regulation was recently observed for *Microcystis* spp., which produces a cyanotoxin and microcystin, and also acts on the epithelial TJ via MLC interference [18]. Additionally, in microcystin-dependent barrier regulation, the specific MLCK inhibitor PIK could mitigate the effects on the TJ. The same specific MLCK inhibitor D-reverse PIK, an amino acid chain and pseudosubstrate of the MLCK [20], was used in the present study for analyzing CDT. It turned out that PIK was also highly effective on CDT. PIK improved the CDT-induced barrier defect, as well as the claudin-4 dislocalization.

From the ruffling of the ZO-1 signal in the TJ together with an increased condensation of F-actin around the TJ observed already at sublethal CDT concentrations, it became obvious that the perijunctional cytoskeleton is the major target of the TJ disruption [41]. While the dysregulation of actin with ribosylation of G-actin by CDTa might be an important hit on the actin cytoskeleton, only the additional change in the phosphorylation status of the epithelial cells by CDTb might have this significant impact on barrier function at low toxin concentrations. Therefore, potential new therapeutic prospects for MLCK inhibition have been proposed for different indications [42], and may also be a target for hypervirulent *C. difficile* infection, which we propose to investigate in the future.

## 4. Materials and Methods

### 4.1. Cell Culture

Human HT-29/B6-GR/MR colon carcinoma cells (a subclone of the HT-29/B6 cell line stably expressing human glucocorticoid α and human mineralocorticoid-receptors, both cell lines were generated in our laboratory of the Clinical Physiology, Charité, Berlin, Germany) [16] were cultivated 25 cm^2^ culture flasks (Corning, Glendale, AZ, USA) in Roswell Park Memorial Institute (RPMI 1640) cell culture medium with GlutaMAX supplement (Gibco, Thermo Fisher Scientific Inc., Waltham, MA, USA) for 7 days at 37 °C in a humidified 5% CO_2_ atmosphere. RPMI 1640 culture media were supplemented with 10% fetal calf serum (FCS, Gibco, Thermo Fisher Scientific Inc., Waltham, MA, USA), 1% penicillin/streptomycin (Gibco, Carlsbad, CA, USA), 300 µg/mL of G418 (Invitrogen, Carlsbad, CA., USA), and 200 µg/mL of hygromycin B (Biochrom GmbH, Berlin, Germany). For experimental use, cells were seeded on polycarbonate filter supports (Millicell-PCF, effective membrane area 0.6 cm^2^, pore size 3 µm, Merck Millipore Ltd., Darmstadt, Germany) and grown to confluence. Experiments were performed 8–9 days after seeding. The medium was changed every 2 days. HT-29/B6 with a stable LSR knockout was created, as previously described by our group, using a sgRNA-CRISPR/ Cas9 vector [43]. These cells were cultured in RPMI 1640 supplemented with 10% fetal calf serum (FCS, Gibco, Thermo Fisher Scientific Inc., Waltham, MA, USA), 1% penicillin/streptomycin (Gibco, Carlsbad, CA, USA), and 1.5 µg/mL of puromycin.

### 4.2. Toxin Treatment and Functional Characterization of Epithelial Barrier Function

The two CDT subunits, CDTa and activated CDTb, were produced at the Institute of Toxicology, Hannover Medical School, Germany. They were generated, as previously described, using an *Escherichia coli* expression system [44]. Cells were treated with 5 ng/mL CDTa and 10 ng/mL CDTb. To determine epithelial barrier function, transepithelial electrical resistance (TER) was measured before and 24 h after CDT treatment. TER values were recorded with chop-stick electrodes and a Volt–Ohm meter (EVOM^3^; World Precision Instruments, Sarasota, FL, USA) under sterile conditions. TER was subsequently corrected for the resistance of an empty filter and calculated assuming a growth area of 0.6 cm^2^. To further characterize the barrier integrity of cell monolayers, epithelial permeability measurements were performed. Thereby, fluorescent markers of different molecular size, fluorescein (332 Da; 100 µM), and FITC-dextran (4 kDa; 200 µM) were used. Fluxes were measured from apical to the basolateral compartment in 12-well plates at 37 °C. Samples were taken from the basolateral side every 15 min up to one hour for fluorescein flux measurements and every 30 min up to two hours for FITC-dextran. The fluorescence signals were measured spectrophotometrically (Tecan GmbH, Maennedorf, Switzerland). Permeability was then calculated as flux/concentration differences.

### 4.3. Cytotoxicity Tests

To detect epithelial apoptosis in CDT-treated HT-29/B6 cell monolayers, terminal deoxynucleotidyl transferase dUTP nick-end labeling (TUNEL) assay (In situ Cell DeathDetection Kit, Roche, Mannheim, Germany) was performed, according to the manufacturer’s instructions. Nuclei were then stained by DAPI (1:1000; Roche AG, Basel, Switzerland). Apoptosis-positive cells were visualized using confocal laser-scanning microscopy (CLSM; Zeiss LSM780, Jena, Germany) and counted per low-power field of approximately 200 to 300 cells. The number of apoptotic events was estimated in three to four randomly picked regions per sample. The ratio of apoptosis was then calculated as the number of TUNEL-positive cells relative to the total number of cells. As a positive control, staurosporine (Sigma Aldrich, St. Louis, MO, USA) was used. To induce apoptosis, a concentration of 1 µM of staurosporine was applied and cells were incubated for 24 h. In further experimental series, cellular cytotoxicity was determined by CyQUANT LDH Cytotoxicity Assay (Invitrogen, Carlsbad, CA, USA), according to the manufacturer’s instructions. Cells of the control group lysed with the kit’s 10× lysis buffer served as an internal positive control for the LDH assay, which equals the maximal LDH release possible. In addition, interferon gamma (IFNγ; 500 or 1000 U/mL) was used as a positive control of cytotoxicity induction and was added in the basolateral compartment for 72 h. Cytotoxicity was calculated using the following formula:

$$\%\;Cytotoxicity=\left(\frac{compound\;treated\;LDH\;activity-spontanous\;LDH\;activity}{Maximum\;LDH\;activity-spontanous\;LDH\;activity}\right)\times 100$$

### 4.4. Western Blot Analysis

Differential expression of TJ proteins was investigated by Western blot analysis. Controls and CDT-treated cell monolayers were washed twice with PBS (Gibco, Life Technologies). Ice-cold whole cell lysis buffer (150 mM NaCl, 10 mM Tris buffer (pH 7.5), 0.5% Triton X-100, 1% SDS, and Complete Protease Inhibitor (Roche AG, Manheim, Germany)) was added, and cells were scraped from the filters. After 30 min of incubation on ice with vortexing in between, cells were centrifuged at 13,000 rpm for 30 min at 4 °C, and the supernatant was collected. Protein concentration was estimated by Pierce bicinchoninic acid (BCA) assay (Thermo Scientific, Waltham, MA, USA), according to the manufacturer’s instructions. Protein samples (20 µg) were then resolved using 12.5% SDS polyacrylamide gels and transferred to a nitrocellulose membrane for 1 h. Afterward, membranes were blocked for 1 h at room temperature with 1% PVP-40 (Polyvinylpyrrolidone; Sigma Aldrich, St. Louis, MO, USA) in TBST/0.05%Tween-20 buffer to avoid unspecific protein signals. Primary antibodies claudin-1 (1:1000 Invitrogen, Carlsbad, CA, USA), claudin-3 (1:1000; Invitrogen), claudin-4 (1:1000; Invitrogen), claudin-5 (1:1000; Invitrogen), tricellulin (1:2000; Invitrogen), occludin (1:1000; Proteintech Rosemont, IL, USA), and anti-GAPDH as a loading control (1:10,000; Sigma Aldrich, St. Louis, MO, USA) were incubated, shaking overnight at 4 °C. Membranes were then incubated with a peroxidase-conjugated secondary antibody, either goat anti-rabbit IgG or goat anti-mouse IgG (Jackson ImmunoResearch, Ely, UK) at room temperature for 2 h. For protein detection, membranes were incubated with SuperSignal West Pico PLUS Stable Peroxide Solution (Thermo Scientific, Waltham, MA, USA). Chemiluminescence was measured using the FUSION FX imaging system (Vilber Lourmat Deutschland GmbH, Eberhardzell, Germany). For quantification of protein bands, densitometric analysis of the Western blots was performed by ImageJ software (Rasband, W. S., ImageJ, National Institute of Health (NIH), Bethesda, MD, USA).

### 4.5. Immunostaining and Super-Resolution STED Microscopy

TJ protein localization in epithelial cell monolayers was analyzed by confocal laser-scanning microscopy (CLSM) or super-resolution stimulated emission depletion (STED) microscopy. Cells grown on 3 µM PCF filters were washed twice with PBS and fixed for 20 min in 2% paraformaldehyde (PFA; Electron Microscopy Science, Hatfield, PA, USA). Primary antibodies were raised against claudin-4 (1:100; Invitrogen), occludin (1:100; Invitrogen), tricellulin (1:400; Invitrogen), and ZO-1 (1:100; BD Biosciences, Franklin Lakes, NJ, USA) and incubated overnight at 4 °C. Secondary antibodies were either conjugated to Alexa Fluor 488 (1:500; Invitrogen) or Alexa Fluor 594 (1:500; Invitrogen) for CLSM or Aberrior STAR RED and Aberrior STAR ORANGE (1:200; Abberior GmbH, Göttingen, Germany). F-actin was stained using Phalloidin DY-647P1 (1:500; Dyomics GmbH, Jena, Germany) or Abberior STAR RED (1:100; Abberior GmbH) and incubated 120 min at room temperature. For CLSM, nuclei were stained with DAPI (1:1000; Roche AG). After incubation, cells on the filters were washed twice with PBS and once with distilled water, then mounted on glass slides using either ProTaq Mount Fluor (Biocyc, Luckenwalde, Germany) or a heated mounting medium (Abberior GmbH) to 65 °C. For STED microscopy, each filter was fixed with a STED-compatible cover slide (Carl Zeiss, Jena, Germany). Localization and subcellular distribution of TJ proteins was determined by confocal laser scanning (Zeiss LSM780, Jena, Germany) and STED (Abberior Facility Line, Abberior GmbH) microscopy.

### 4.6. Inhibitor Studies

Eight-day-old cells grown on filter supports were placed in a 12-well plate. For the inhibition of apoptosis, the pan-caspase inhibitor Q-VD-OPh hydrate (10 μM; Calbiochem, San Diego, CA, USA) dissolved in DMSO was applied. A medium containing the inhibitor was added on the basal side 1 h before the toxin treatment. To study MLCK inhibition, the culture medium was supplemented with the specific MLCK pseudosubstrate inhibitor D-reverse PIK (200 μM) [20]. The D-reverse PIK was added apically, 30 min prior to CDT treatment.

### 4.7. Statistics

Data are expressed as mean values ± standard error of the mean (SEM). Statistical analysis was performed with GraphPad Prism (GraphPad Software version 7, San Diego, CA, USA). *p*-values were calculated either by Student’s *t*-test with Bonferroni correction or by one-way ANOVA with Bonferroni correction. *p* < 0.05 was considered statistically significant.

## Data Availability

The data presented in this study are available in this article.
